# Mesenchymal stem cells promote cisplatin resistance in non-small cell lung cancer through IL-6/MEK-ERK/macrophages axis: construction of prognostic signature and experimental investigation

**DOI:** 10.3389/fphar.2026.1793151

**Published:** 2026-04-01

**Authors:** Xinchun Ma, Zhihui Zhang, Ni Liu, Xiaoling Shang, Shumin Yuan, Chenxi Wei, Xuan Sun, Zixu Wang, Xiuwen Wang, Yanguo Liu

**Affiliations:** 1 Department of Medical Oncology, Qilu Hospital of Shandong University, Jinan, China; 2 Cheeloo College of Medicine, Shandong University, Jinan, China; 3 Department of Radiation Oncology, National Cancer Center/National Clinical Research Center for Cancer/Cancer Hospital, Chinese Academy of Medical Sciences and Peking Union Medical College, Beijing, China; 4 Department of Oncology, Nanfang Hospital, Southern Medical University, Guangzhou, Guangdong, China

**Keywords:** cisplatin, interleukin-6, macrophages, mesenchymal stem cells, tumor microenvironment

## Abstract

**Background:**

Cisplatin is a primary treatment for non-small cell lung cancer (NSCLC), however, cisplatin resistance develops readily, leading to tumor progression. Mesenchymal stem cells (MSCs) distributed in the tumor microenvironment (TME), yet the effects and mechanisms of cisplatin on MSCs and their subsequent impact on TME remains unclear.

**Methods:**

The MSC-related prognostic signature was developed through Cox and LASSO regression analysis. Validation was performed by survival analysis, ROC analysis and nomogram construction. TME evaluation was carried out by GSEA and immune infiltration analysis. The effect of cisplatin on MSCs was investigated by polymerase chain reaction (PCR) array, qRT-PCR, ELISA and Western blot. The migration of RAW 264.7 macrophages was analyzed by transwell assays and the polarization was analyzed by flow cytometry.

**Results:**

High-risk of MSC-related prognostic signature (PDGFB, ANPEP, CD40) was significantly linked to poor prognosis in patients under cisplatin treatment for NSCLC and other cancers. Patients with high IL-6 expression demonstrated poor response to cisplatin therapy. MSCs were linked with an immunosuppressive TME characterized by macrophage infiltration, particularly M2 macrophages. Cisplatin upregulated IL-6 expression in MSCs via the MEK-ERK pathway. The ability of MSCs to promote RAW 264.7 macrophages recruitment and polarization was enhanced by cisplatin.

**Conclusion:**

We established MSC-related prognostic signature for cisplatin therapy in NSCLC. MSCs and IL-6 were associated with cisplatin resistance and macrophages infiltration. Thus, MSCs and IL-6 were potential targets for NSCLC therapeutic intervention.

## 1 Introduction

Cancer is a globally prevalent malignant disease with a high rate of mortality, among which lung cancer is the leading reason of cancer-related deaths (Bray et al., 2024). Cisplatin-based doublet chemotherapy continues to be the mainstay of treatment for cancer patients, especially in non-small cell lung cancer (NSCLC) without driver gene mutations (Meyer et al., 2024; Jeon et al., 2025). Traditionally, cisplatin is thought to exert its cytotoxic activities by crosslinking DNA and inhibiting mitosis (Zhang et al., 2022). However, studies have revealed that cisplatin has important immunomodulatory effects on tumor pathogenesis and progression by regulating immune cell recruitment and promoting cytokine or chemokine expression in the tumor microenvironment (TME) (Li et al., 2025; Meng et al., 2024).

TME consists of a complex mixture of cells that fulfill different functions, including immune cells, malignant cells and stromal cells, also mesenchymal stem cells (MSCs) (Zhou et al., 2023). During tumor progression, dynamic interactions among tumor cells, stromal cells and immune cells collectively shape the immunological landscape, which critically determines patient survival rates and therapeutic efficacy (Ke et al., 2025; Ke et al., 2023). MSCs support tissue regeneration, restore the microenvironment following damage by chemotherapy, or enhance cell migration into tumors (Galderisi et al., 2022; Starska-Kowarska, 2024; Wang M. Q. et al., 2023). This specific migration to tumor sites is guided by chemokines (Spaeth et al., 2008; Morein et al., 2020). MSCs secrete various cytokines to attract stromal and immune cells to the TME. Circulating macrophages and monocytes are recruited into tumor tissues, where they predominantly differentiate into tumor-associated macrophages (TAMs), with activated tumor-promoting M2 phenotypes (Zhang and Sioud, 2023; Gao et al., 2022). TAMs contribute to immunosuppression, protect cancer stem cells, stimulate cancer cell proliferation and consequently promote cancer progression (Chu et al., 2024; Chen et al., 2022). TAMs density is closely related with poor prognosis in lung cancer and TAMs mediate chemosensitivity in breast and pancreatic cancer (Hwang et al., 2020; Ye et al., 2021; D'Errico et al., 2019).

Interleukin (IL)-6 is a multifunctional cytokine that has been shown to be related to inhibition of tumor cell apoptosis, promotion of tumor survival, development and metastasis, regulation of stem cell self-renewal and tumor-associated immunosuppression (Thuya et al., 2025; Taniguchi and Karin, 2014; Wu SS. et al., 2025; Chen et al., 2024). There is evidence that elevated IL-6 is linked to poor prognosis as well as drug resistance in head and neck carcinoma (HNSC) and breast cancer (De La Cruz-Vargas et al., 2025; Song et al., 2024; Spanko et al., 2021; Radharani et al., 2022).

MSCs can be activated by chemotherapeutics, including cisplatin, and further induce resistance to chemotherapy (Yu et al., 2021; Liu et al., 2021; Wang Q. Q. et al., 2023). However, the underlying mechanisms remain partially understand. Our study developed the MSC-related prognostic signature for cisplatin therapy in NSCLC and other cancers. And we analyzed immune infiltration characteristics in TME, identifying macrophages as a key component. We investigated the impact of cisplatin on MSCs IL-6 secretion and investigated its role in macrophages recruitment and polarization. Our study suggests the potential of MSC-related prognostic signature in cisplatin therapy and provided clues about MSCs in promoting suppressive TME and cisplatin resistance.

## 2 Methods

### 2.1 Data preparation and processing

All datasets utilized in this study were sourced from the Cancer Genome Atlas (TCGA) and the Gene Expression Omnibus (GEO). Transcriptome and corresponding clinical information of lung squamous cell carcinoma (LUSC) patients and lung adenocarcinoma (LUAD) patients treated with cisplatin were downloaded from TCGA database. After removing normal samples and cases with missing survival information, 156 NSCLC patients receiving cisplatin treatment were finally included. Similarly, data and information of 138 cervical squamous cell carcinoma and endocervical adenocarcinoma (CESC), 94 HNSC and 112 ovarian serous cystadenocarcinoma (OV) patients treated with cisplatin were also obtained from the TCGA. GSE14814 contains microarray datasets and clinical information of 71 NSCLC patients treated with cisplatin. Gene expression was normalized using the Transcripts Per Million (TPM). The R package “Combat” was used to eliminate the batch effect.

### 2.2 Identification of MSC biomarkers

Biomarker genes of human MSCs were provided by the CellMarker2.0 database. We screened these biomarkers for those that were experimentally validated or appeared more than once in sequencing analysis (Supplementary File 1). Marker genes were input into the STRING database to obtain the protein interaction network relationship. The protein-protein interaction (PPI) network was constructed by the Cytoscape software. The R package “clusterProfiler” was used to conduct Gene ontology (GO) function enrichment in biological process (BP), cellular component (CC), and molecular function (MF) terms and kyoto encyclopedia of genes and genomes (KEGG) pathway analysis (p < 0.05 was considered significance) to identify biological functions, metabolic processes and molecular mechanisms, in which the MSC markers were involved.

### 2.3 Construction of MSC-related prognostic signature

The 156 TCGA-NSCLC patients receiving cisplatin treatment were designated as the training cohort, while 71 cisplatin-treated NSCLC patients from the GSE14814 dataset were served as the validation cohort. Univariate Cox regression analysis and least absolute shrinkage and selection operator (LASSO) regression were applied via R package “survival” and “glmnet”. And then the stepwise multivariate Cox regression was performed for calculating the regression coefficient. The riskscores of MSCs-related risk model was calculated as: Riskscore = Σ (Gene Expression_i_ × Coefficent_i_).

### 2.4 Validation of MSC-related prognostic signature

The “survival” package was utilized to plot Kaplan–Meier (K-M) survival curves for TCGA-NSCLC training cohort and GSE14814 validation cohort, as well as the TCGA-CESC, TCGA-HNSC and TCGA-OV cohorts. The “timeROC” package was utilized to perform the time-dependent receiver operating characteristic (ROC) analysis. The area under the curve (AUC) and 95% confidence interval were employed to evaluate the predictive accuracy. Afterwards, we integrated the clinical features with MSC riskscores through the Cox regression analysis. The “rms” R package was used to construct the nomogram and the clinical utility was evaluated through the consistency index (C-index) and the calibration curves.

### 2.5 Gene set enrichment analysis (GSEA)

The R package “clusterProfiler” was utilized to perform GSEA analysis. the reference gene sets were “c2.cp.kegg.Hs.symbols.gmt” and “c5.go.v7.4.symbols.gmt”. The top five enriched biological pathways were identified for display.

### 2.6 Immune infiltration analysis

Infiltration of immune cells of each patient were calculated by 7 algorithms (including CIBERSORT, CIBERSORT-ABS, MCP-counter, xCell, EPIC, TIMER and QUANTISEQ). The correlation between MSC riskscores and immune cell scores was analyzed by Spearman. The single-sample Gene Set Enrichment Analysis (ssGSEA) was evaluated by the “GSVA” package to calculate immune cell abundance levels and distinct between risk groups was analyzed by Wilcoxon test. The immune scores and stroma scores were employed by Estimation of STromal and Immune cells in MAlignant Tumor tissues using Expression data (ESTIMATE) algorithm.

### 2.7 Cell culture

Mouse and human bone marrow MSCs (Procell, Wuhan, China) were cultured in high-glucose Dulbecco’s modified Eagle’s medium (DMEM; Gibco, Carlsbad, CA, USA) with 10 ng/mL basic fibroblast growth factor recombinant protein (b-FGF; Invitrogen, Carlsbad, CA, USA), 10% fetal bovine serum (FBS; Corning Inc., Herndon, VA, USA), 2 mmol glutamine, 100 μg/mL streptomycin and 100 U/ml penicillin. Lewis lung cancer cells (LLC cells, American Type Culture Collection, Manassas, VA, USA) and RAW 264.7 macrophages (Chinese Academy of Medical Sciences, Beijing, China) were cultured in DMEM with 10% FBS, 100 μg/mL streptomycin and 100 U/ml penicillin (Yu et al., 2025). Cells were cultured at 37 °C under 5% CO_2_ atmosphere.

### 2.8 Polymerase chain reaction (PCR) array and screening of members of the IL family

MSCs were exposed to 5 µmol cisplatin (Sigma-Aldrich, Munich, Germany) for 6 h, with the same amount of normal saline (NS) as control. After 6 h, we exchanged the medium with fresh medium without cisplatin to remove the cisplatin. At 0, 6 and 24 h, the MSCs were lysed, the RNA was isolated, and the genes encoding the members of the IL family were amplified according to the PCR array instructions (Qiagen, Duesseldorf, Germany).

### 2.9 Quantitative real-time (qRT)-PCR

MSCs and LLC cells were exposed to cisplatin for 6, 12,18, and 24 h (same amount of NS as control), as above. The MSCs were lysed and RNA was isolated according to the RNA isolation instructions (Qiagen, Duesseldorf, Germany). mRNA expression was analyzed by SYBR Green PCR Master Mix (Takara Bio, Dalian, China) on Light Cycler 480 Real-Time PCR System (Roche, Basel, Switzerland). Mouse IL-6 forward primer, 5′-TCC​AGT​TGC​CTT​CTT​GGG​AC-3′; mouse IL-6 reverse primer, 5′-AGA​CAG​GTC​TGT​TGG​GAG​TG-3′; mouse β-actin forward primer, 5′-TAA​GAG​GAG​GAT​GGT​CGC​GT-3′; mouse β-actin reverse primer, 5′-AAG​TCA​GTG​TAC​AGG​CCA​GC-3′; human IL-6 forward primer, 5′-CCA​GCC​TGC​TGA​CGA​AGC-3′; human IL-6 reverse primer, 5′-TCA​GGC​TGG​ACT​GCA​GGA​AC-3′; human β-actin forward primer, 5′-CAC​TCT​TCC​AGC​CTT​CCT​TCC-3′; human β-actin reverse primer 5′-GCA​CTG​TGT​TGG​CGT​ACA​GG-3′.

### 2.10 Enzyme-linked immunosorbent assay (ELISA)

MSCs were pre-incubated with the mitogen-activated protein kinase (MEK)-extracellular signal-regulated kinase (ERK) pathway inhibitor PD98059 (Selleck Chemicals, Houston, TX, USA) at a concentration of 10 µmol for 30 min, with same amount of NS as control. The cells were then treated with 5 µmol cisplatin for 6 h. The fresh medium (containing PD98059) was exchanged to remove the cisplatin while keeping the effect of PD89059. At 6, 12,18, and 24 h afterward, the medium was harvested to perform IL-6 ELISA kit (R&D Systems, Minneapolis, MN, USA).

### 2.11 Western blot analysis

MSCs were harvested in lysis buffer supplemented with a protease and phosphatase inhibitor cocktail (Zhang et al., 2025). The resulting lysates were resolved on 10% polyacrylamide gel electrophoresis (PAGE) gels (Dakewe Biotech Co., Shenzhen, China) (30 μg/mL) and transferred onto nitrocellulose membranes (Whatman, Maidstone, United Kingdom). 5% nonfat milk was used to block membranes. They were then hybridized with p-ERK1/2, t-ERK1/2, and glyceraldehyde 3-phosphate dehydrogenase (GAPDH) antibodies (1:1000; Cell Signaling Technology, Danvers, MA, United States) and then washed. Horseradish peroxidase-conjugated secondary antibodies (1:10000; Cell Signaling Technology, Danvers, MA, United States) were used to incubate membranes. Membranes were visualized using enhanced chemiluminescence reagents (GE Healthcare Life Sciences, Marlborough, MA, United States).

### 2.12 Transwell assays

To investigate the function of MSCs, we carried out transwell assays (8.0 µm, Corning, NY, United States) to assess the macrophages recruitment. The lower chambers contained 600 µL different MSCs conditioning medium according to the following groups: 1) negative control group (NC, DMEM); 2) IL-6 group (0.25 ng/mL IL-6 recombinant protein in DMEM); 3) MSCs control group (2 × 10^5^ MSCs in normal medium); 4) cisplatin group (MSCs after abovementioned cisplatin treatment); 5) cisplatin + PD98059 group (MSCs after abovementioned cisplatin and PD98059 treatment); and 6) cisplatin + IL-6 antibody group (identical to the cisplatin group but with 0.03 μg/mL IL-6 antibody). Subsequently, 100 µL RAW 264.7 macrophages (8 × 10^3^/100 µL in each chamber) were added to the upper chamber with serum-free medium. After incubation for 24 h, the macrophages in the upper chambers were removed. Macrophages that migrated to the lower surface were fixed in 70% ethanol for 20 min, then washed and stained with 0.2% crystal violet for 15 min.

### 2.13 Flow cytometry

RAW 264.7 macrophages treated with different MSCs conditioning medium for 48 h were adjusted to 1 × 10^6^ cells in 100 µL PBS. Cells were stained with TruStain FcX antibody (1:200, Biolegend, San Diego, CA, United States) for 10 min at 4 °C and then cells were fixed and permeabilized using True-Nuclear™ Transcription Factor Buffer Set (Biolegend), and stained with anti-CD206-APC and anti-arginase 1 (Arg-1)-FITC (1:100, Biolegend) for 30 min at 4 °C. Flow cytometry data were analyzed by FlowJo V.10.1 software.

### 2.14 Statistical analysis

Statistical analyses and graphs were performed using R Software 4.4.3 and GraphPad Prism 9. The t-test and one-way ANOVA were applied for comparison of groups in experiments. p < 0.05 was considered statistically significant.

## 3 Results

### 3.1 Identification of MSC biomarkers and construction of MSC-related prognostic signature

**FIGURE 1:**
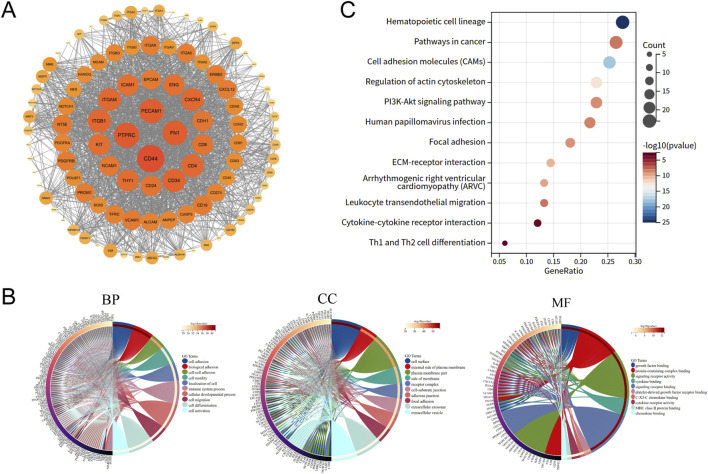
Identification of MSC biomarker genes and enrichment analysis. **(A)** PPI network of MSC biomarkers. **(B)** GO function enriched in MSC markers. **(C)** KEGG pathways enriched in MSC markers.

A total of 588 terms of biomarker records of MSCs were downloaded and 105 genes were yielded after screening and integration. In order to visualize the interrelationships among these biomarkers, a PPI network with 103 interacting proteins and 1800 interaction pairs was constructed (Figure 1A). The GO function enrichment and KEGG pathway analysis revealed that these genes were enriched in “focal adhesion”, “ECM-receptor interaction”, “pathways in cancer”, “cytokine-cytokine receptor interaction” “extracellular exosome”, “cell activation”, “cell migration”, “immune system process” and “Th1 and Th2 cell differentiation” (Figures 1B,C). These were consistent with the biological characteristics of MSCs.

**FIGURE 2:**
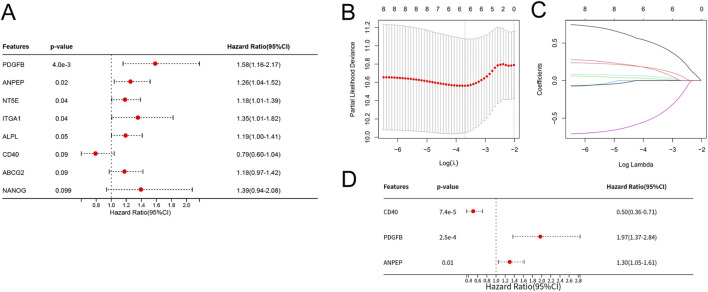
Construction of a MSCs-related prognostic signature for NSCLC patients treated with cisplatin. **(A)** Univariate cox regression analysis of MSC biomarkers. **(B,C)** LASSO regression of MSC biomarkers. **(D)** Multivariate Cox regression.

According to univariate Cox regression analysis, eight genes were identified as prognostic factor for cisplatin treatment in NSCLC (p < 0.1) (Figure 2A). LASSO regression analysis was employed and six candidate genes were selected (Figures 2B,C). Subsequently, multivariate Cox analysis was carried out to identify final genes and finally, the signature based on MSC biomarkers and worked for cisplatin-treated NSCLC patients was constructed (Figure 2D). The riskscore calculation formula is as follows: Riskscore = 0.66 × PDGFB +0.23 × ANPEP - 0.70 × CD40.

Among the three signature genes, HR of MSCs positive biomarkers PDGFB and ANPEP were more than 1, while HR of MSCs negative biomarker CD40 was less than 1, indicating that high MSCs expression in the TME may be associated with poor efficacy of cisplatin.

### 3.2 The MSC-related prognostic signature robust predictive accuracy for patients receiving cisplatin therapy

**FIGURE 3:**
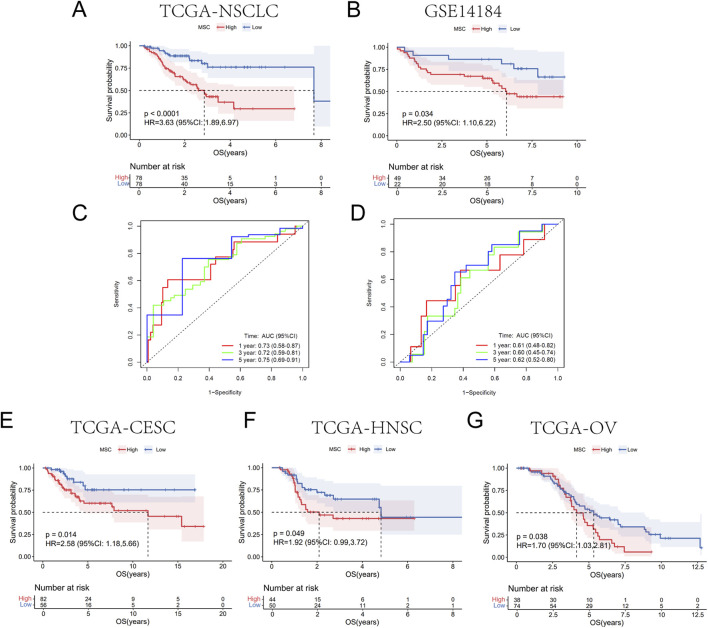
Validation of MSC-related prognostic signature for evaluating cisplatin efficacy. **(A,B)** K-M curves for OS in training **(A)** and validation cohort **(B)**. LUSC and LUAD patients undergoing cisplatin therapy. **(C,D)** ROC curves for 1-, 3-, and 5-year survival. **(E–G)** OS survival in CESC **(E)**, HNSC **(F)** and OV **(G)** cohorts.

K-M survival results suggested that patients in MSC high-risk group owned significantly shorter OS in both training cohort (HR = 3.63, p < 0.0001) and validation cohort (HR = 2.50, p = 0.034) (Figures 3A,B). Furthermore, ROC curve results suggested that the AUC for predicting 1, 3, 5-year survival were 0.73, 0.72, 0.75 in the training cohort and 0.61, 0.60, 0.62 in the validation cohort, respectively (Figures 3C,D). These results revealed that NSCLC patients with high MSC risk were significantly related with poor response to cisplatin therapy and the signature had excellent predictive capability. Otherwise, we validated the efficacy of MSC-related prognostic signature in other tumors to verify the predictive power. Patients with high MSC risk also displayed shorter OS in TCGA-CESC, TCGA-HNSC and TCGA-OV cisplatin-treated cohorts (Figures 3E–G).

**FIGURE 4:**
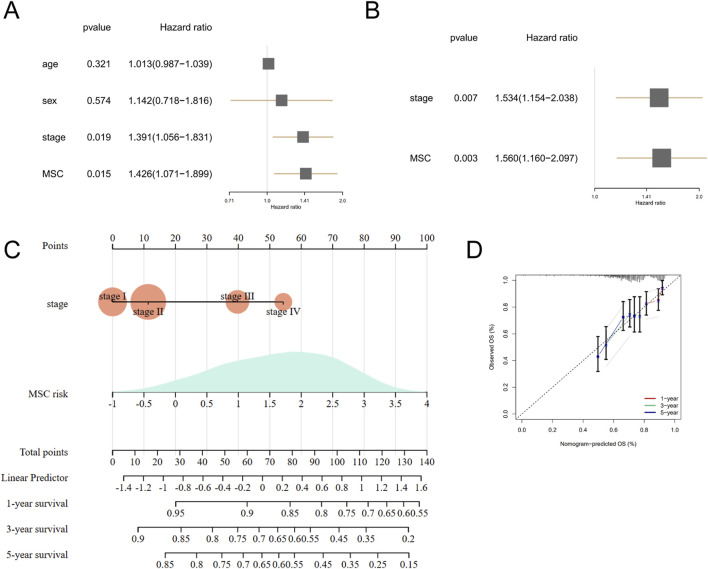
Construction of MSCs-related nomogram for predicting the efficacy of cisplatin efficiency. **(A,B)** Univariate Cox regression and multivariate Cox regression for clinical characteristics and MSC riskscores. **(C)** Nomogram for cisplatin efficacy. **(D)** Calibration curve.

For further verification, univariate Cox regression analysis on clinical characteristics revealed that tumor stage (HR = 1.391, p = 0.019) and MSCs-related risk model riskscore (HR = 1.426, p = 0.015) were significantly linked to prognosis of NSCLC patients with cisplatin treatment (Figure 4A). Multivariate Cox regression demonstrated that tumor stage (HR = 1.534, p = 0.007) and MSC riskscores (HR = 1.560, p = 0.003) were independent prognostic factors for cisplatin therapy in NSCLC (Figure 4B). Subsequently, we constructed a nomogram that integrated the riskscore of MSCs-related risk model with tumor stage to predict the efficacy of cisplatin in NSCLC patients (Figure 4C). The C-index for the nomogram yielded a value of 0.62 and the calibration curves showed good predictive performance indicating its reliable predictive capability regarding cisplatin efficacy (Figure 4D).

### 3.3 The MSC-related signature was correlated with substantial infiltration of macrophages in TME

**FIGURE 5:**
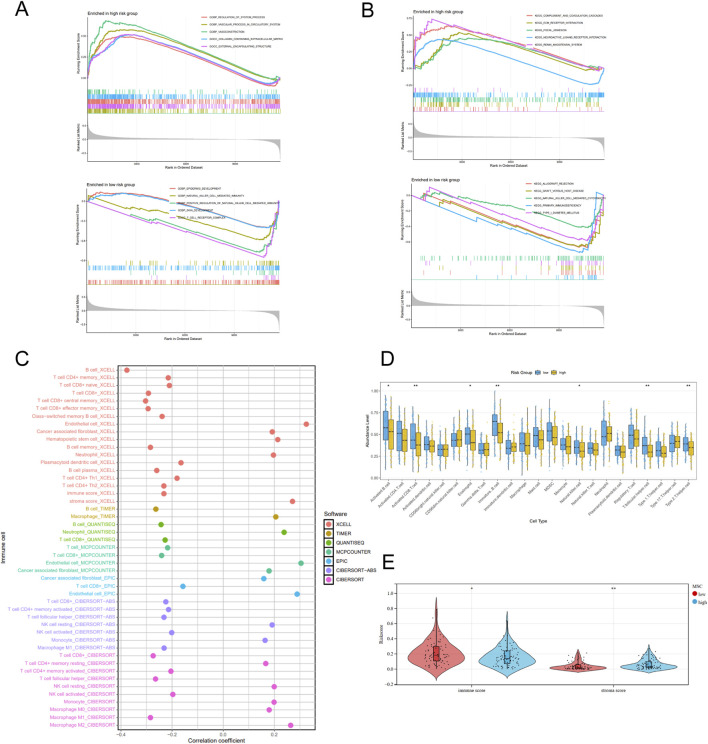
TME differences between two risk groups. **(A,B)** GSEA for GO **(A)** and KEGG **(B)**. **(C)** The correlation between MSC-related signature and immune cell subtypes through multiple algorithms. **(D)** The difference of immune cell abundance levels calculated by ssGSEA. **(E)** The difference of stromal scores between two risk groups.

The TME was commonly considered to be related with the development and progression of lung cancer and patients’ prognosis and response to cisplatin. GSEA revealed distinct pathways between high- and low-risk groups. The high-risk group was connected with collagen containing extracellular matrix, vasoconstriction, ECM receptor interaction and focal adhesion, while T cell receptor complex, natural killer (NK) cell mediated immunity, NK cell mediated cytotoxicity were enriched in the low-risk group (Figures 5A,B). These findings suggested different molecular mechanisms underlying MSC-related prognostic signature.

Through analysis of the immune microenvironment, we revealed a complex immune infiltration landscape. Results obtained from multiple algorithms indicated positive correlations between MSC-related signature and resting NK cells, resting memory CD4^+^ T cells, macrophages, M2 macrophages, monocytes, neutrophils and cancer associated fibroblasts (CAFs), endothelial cells and mast cells. Furthermore, the signature was significantly negatively associated with B cells, CD8^+^ T cells, central and effector memory CD8^+^ T cells, activated NK cells, helper CD4^+^ T (Th) cells, activated memory CD4^+^ T cells and M1 macrophages (Figure 5C). ssGSEA immune analysis revealed decreased infiltration levels of activated B cells, activated CD8^+^ T cells, NK cells and Th cells in the high-risk group (Figure 5D). Subsequently, estimate analysis demonstrated significantly lower immune scores and higher stroma scores in high-risk patients (Figure 5E). These results indicated that MSCs were associated with an immunosuppressive TME characterized by reduced infiltration of activated immune cells and increased TAMs and stromal cells, which may further contribute to cisplatin resistance.

### 3.4 Cisplatin upregulated IL-6 expression in MSCs

**FIGURE 6:**
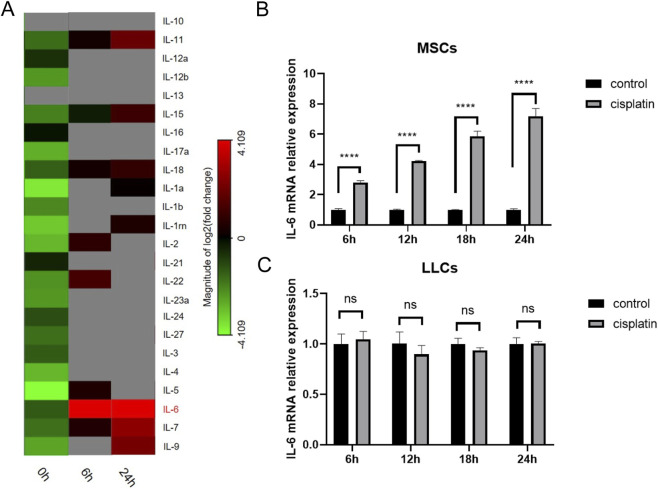
Cisplatin specifically upregulated IL-6 expression in MSCs. **(A)** PCR array results revealed IL family members expression by MSCs after 6-h and 24-h cisplatin treatment. **(B,C)** IL-6 mRNA expression levels of MSCs **(B)** and LLC cells **(C)** at 6, 12, 18, and 24 h after cisplatin treatment assayed by PCR. ****p < 0.0001.

The TME was commonly considered to be related with the efficacy of chemotherapy, thus the poor prognosis of NSCLC patients may be associated with regulation of MSCs in TME by cisplatin. To investigate the impact of cisplatin on cytokine secretion of MSCs, we detected the expression of members of the IL family by PCR array. Among the IL family members, IL-6 expression was most obviously upregulated at 6 h (18.46-fold) and 24 h (21.22-fold) after cisplatin treatment (Figure 6A). The increased IL-6 mRNA level of MSCs at 6, 12, 18, and 24 h after cisplatin treatment further proved the accuracy of the PCR array results (Figure 6B). There was no significant difference in LLC cells IL-6 expression after cisplatin stimulation (Figure 6C).

### 3.5 Cisplatin activated IL-6 expression through the MEK-ERK pathway

**FIGURE 7:**
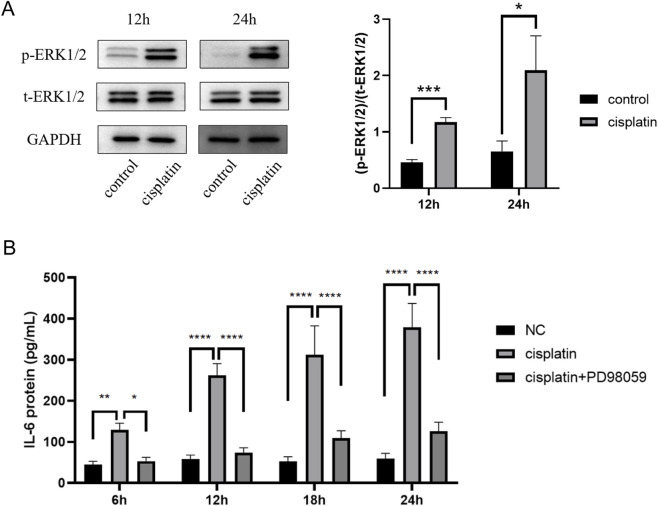
Cisplatin stimulated IL-6 secretion through activating the MEK-ERK pathway. **(A)** Western blot showed p-ERK1/2 and t-ERK1/2 expression at 12 and 24 h after treatment of cisplatin according to Western blotting. **(B)** ELISA results revealed IL-6 protein expression of MSCs at 6, 12, 18, and 24 h after treatment of cisplatin plus PD98059. *p < 0.05, **p < 0.01, ***p < 0.001, ****p < 0.0001.

To investigate the mechanism by which cisplatin induced changes in IL-6 levels in MSCs, we validated the MEK-ERK pathway activation in MSCs after cisplatin treatment. The increased p-ERK1/2 to t-ERK1/2 ratio suggested the activation of MEK-ERK pathway (Figure 7A).

To further investigate the correlation between the IL-6 expression and the MEK-ERK pathway, we used PD98059, a MEK-ERK pathway inhibitor. The results revealed that PD98059 significantly inhibited the IL-6 expression enhancement after cisplatin treatment (Figure 7B; Supplementary Figures S1A, B). Collectively, these fundings indicated that cisplatin activated the MEK-ERK pathway to promote IL-6 expression by MSCs.

### 3.6 Cisplatin enhanced the ability of MSCs to promote macrophages recruitment and M2 polarization via IL-6

**FIGURE 8:**
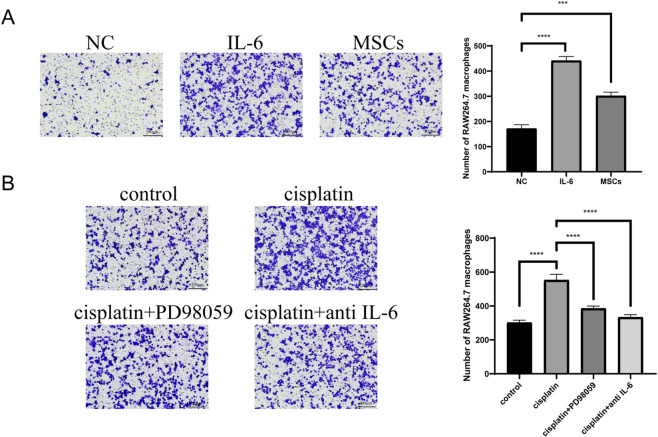
Cisplatin enhanced the ability of MSCs to recruit macrophages. **(A)** Transwell assays showed RAW 264.7 macrophages recruitment in negative control group (NC), IL-6 group and MSCs control group. **(B)** Transwell assays showed RAW 264.7 macrophages recruitment in MSCs control group, cisplatin group, PD98059 group and IL-6 antibody group. ***p < 0.001, ****p < 0.0001.

**FIGURE 9:**
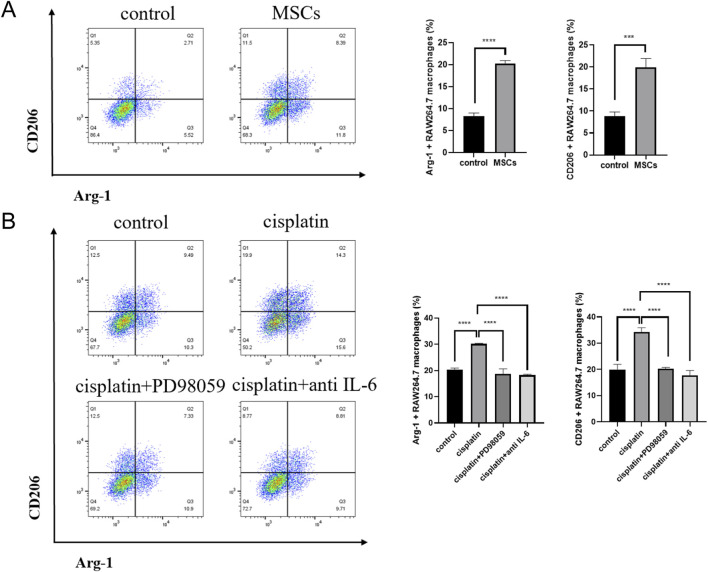
Cisplatin enhanced the ability of MSCs to promote M2 polarization. **(A)** Flow cytometry showed M2 polarization markers expression of RAW 264.7 macrophages in negative control group (NC) and MSCs control group. **(B)** Flow cytometry showed M2 polarization markers expression of RAW 264.7 macrophages in MSCs control group, cisplatin group, PD98059 group and IL-6 antibody group. ***p < 0.001, ****p < 0.0001.

To investigate the function of MSCs on macrophages recruitment, the transwell assays were carried to measure migration ability of RAW 264.7 macrophages attracted by MSCs. The results suggested that IL-6 and MSCs significantly recruit macrophages (Figure 8A). Cisplatin significantly increased RAW 264.7 macrophages recruitment triggered by MSCs and this was inhibited by MEK-ERK pathway inhibitor. When IL-6 protein was neutralized by IL-6 antibody, the number of RAW 264.7 macrophages attracted by MSCs was significantly reduced (Figure 8B). Similarly, MSCs significantly upregulated expression of M2 polarization markers CD206 and Arg-1 in RAW 264.7 macrophages (Figure 9A). And increased CD206 and Arg-1 expression triggered by MSCs was inhibited by PD98059 and IL-6 antibody (Figure 9B).

### 3.7 IL-6 was associated with cisplatin resistance

**FIGURE 10:**
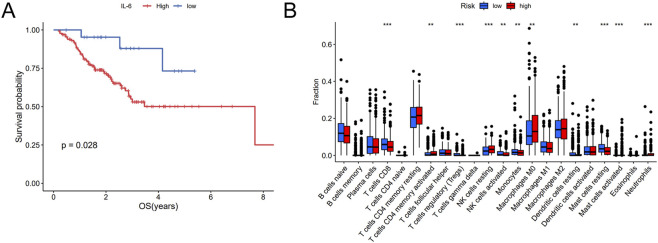
Analysis of survival and immune infiltration in relation to IL-6 expression. **(A)** K-M analysis for OS of IL-6 in NSCLC patients undergoing cisplatin therapy. **(B)** Box plot comparing the abundances of 22 immune cells. **p < 0.01, ***p < 0.001.

K-M curves revealed that patients with high IL-6 expression owned spoor cisplatin efficacy (p = 0.028) (Figure 10A). Notably, immune cell subsets analysis performed by CIBERSORT algorithm revealed increased proportions of M0 macrophages while reduced proportions of CD8^+^ T cells and activated NK cells in patients with high IL-6 expression (Figure 10B). Therefore, we demonstrated that IL-6 was pivotal in cisplatin treatment of NSCLC.

## 4 Discussion

Cisplatin causes DNA damage, obstructs DNA repair mechanisms, interferes with cell division, and subsequently leads to apoptosis (Romani, 2022). The direct destruction of tumor cells by cisplatin has been well studied. However, the impact of cisplatin on MSCs’ ability to recruit immune cells to the tumor site remains unclear.

Previous research revealed that MSCs exhibit immunosuppressive properties, indicating that MSCs have considerable therapeutic potential in treatment of various immune disorders, especially cancers (Hazrati et al., 2023; Tang et al., 2024). We collected cisplatin therapy cohorts and constructed a unique prognostic signature utilizing MSC biomarker genes in patients treated with cisplatin from TCGA-NSCLC cohorts. Studies have suggested that platelet-derived growth factor beta polypeptide (PDGFB) was a significant negative prognostic factor for NSCLC and the expression was decreased after cisplatin combination therapy (Wang and Ren, 2024; Xiao et al., 2024). Alanyl aminopeptidase (ANPEP) was reported to promote liver cancer growth and metastasis (Wu Y. et al., 2025). CD40 agonists increased number and function of dendritic cells in pancreatic ductal adenocarcinoma (Hogg et al., 2025). The reliability and accuracy of signature were validated in GSE14814. Notably, the risk model showed consistent performance across TCGA-CESC, TCGA-HNSC and TCGA-OV cisplatin therapy cohorts, demonstrating the ubiquitous applicability of MSC-related prognostic signature. Subsequently, this study provide evidence that MSCs might be a pragmatic tool to anticipate the treatment efficacy of cisplatin.

According to our findings, significant variations were observed in the abundance of immune cells infiltration between two risk groups. The MSC high-risk was linked with low infiltration of activated CD8^+^ T cells, NK cells and CD4^+^ cells, while high infiltration of macrophage, particularly M2 macrophage and CAFs, pointing towards an immunosuppressive TME. MSCs are involved in immunomodulatory activity, with pro- and anti-tumorigenic functions due to the interactions between MSCs and the inflammatory microenvironment (Harrell et al., 2021). They influence the immune cell functions through direct contact and local microenvironmental factors, mainly via cytokines secretion (Song et al., 2020).

Chemotherapy resistance is an important hindrance to achieving therapies in patients and is the crucial cause of death in most progressive stage cancers (Vasan et al., 2019). Recent studies have demonstrated that MSCs can induce resistance of cancer cells to chemotherapy drugs *in vitro* and *in vivo*. MSCs were reported to induce gastric cancer (GC) cells resistance to cisplatin/vincristine through the upregulation of miR-301b-3p and downregulation of TXNIP (Zhu et al., 2023). Wu et al. (2020) observed that MSCs improved chemo-resistance in GC cells through inducing LncRNA HCP5 and promoting fatty acid oxidation. Furthermore, the upregulation of HIF1A-AS3 reprogramed MSCs to the CAF phenotype, consequently enhancing the resistance of GC cells to chemotherapy (Xu et al., 2025). Previous studies have demonstrated the impact of MSCs on tumor chemotherapy resistance.

The immunosuppressive function of MSCs has been demonstrated to involve nitric oxide, IL-10 and indoleamine 2.3-dipxygenase, but the exact mechanisms remain unclear (Su et al., 2023; Shephard et al., 2022; Hui et al., 2025; Naji et al., 2019). In our study, we used PCR array to screen for the expression of the IL family members by MSCs and the results suggested that cisplatin most significantly upregulated IL-6. We wondered whether the IL-6 upregulation effect of cisplatin differs between cell types. The difference in the response to cisplatin between MSCs and LLC cells demonstrated the different responses to chemotherapy by stromal and tumor cells. This phenomenon indicates the different effects of cisplatin on the microenvironment, and acknowledgement of these different effects may represent a breakthrough in the research on lung cancer progression, recurrence and drug resistance.

Previous studies showing that cisplatin activated the ERK pathway and the ERK pathway can promote IL-6 synthesis (Wu S. S. et al., 2025; Suman et al., 2024; Ko et al., 2025; Chung et al., 2021; Riegel et al., 2021). Here, we provided further evidence linking these two processes. IL-6 expression of MSCs was upregulated by cisplatin and significantly reduced after MEK-ERK pathway inhibitor treatment. Moreover, it has been indicated that IL-6 stimulated the MEK-ERK pathway in turn in tumor cells, causing tumor cell proliferation, survival, metastasis, and chemotherapy resistance (Neurath and Finotto, 2011; Nyati et al., 2021; Qi et al., 2024; Huang et al., 2024; Wu et al., 2023). Thus, we hypothesize that cisplatin triggers the loop between the MEK-ERK pathway and IL-6, resulting in further IL-6 upregulation, which may partly explain the high rate of cisplatin resistance among patients.

High IL-6 expression was also related to poor efficacy of cisplatin, increased level of macrophages infiltration and decreased level of CD8^+^ T cells. These findings indicated IL-6 may be pivotal in the response to cisplatin in the TME. The ability of MSCs to recruit macrophages was enhanced by cisplatin and attenuated by IL-6 antibody, indicating that MSCs recruited macrophages to the TME via IL-6.

TAMs have been shown to act as pro-tumor factors by activating angiogenesis, immune suppression, and anti-apoptotic programs in both solid and hematological malignancies to promote tumor proliferation (Gao et al., 2022; Sharen et al., 2022). And depletion of TAMs has been shown to relieve immunosuppression and improve chemotherapeutic responses (Basak et al., 2023; Zhu et al., 2014; Wesolowski et al., 2019). IL-6 secreted by MSCs in the TME specifically recruit macrophages to the tumor site, which may lead to the development of pro-tumorigenic microenvironment and drug resistance.

Stratification based on clinical manifestations and disease staging enables the development of personalized treatment regimens for distinct subgroups, achieving precision medicine. This study suggested that MSCs are a potential therapeutic target for future treatment strategies. Nevertheless, there are still limitations for current study. More cisplatin treatment cohorts are needed to validate the MSC-related prognostic signature and *in vivo* experiments are required for verifying the mechanisms.

## 5 Conclusion

This study represented that MSC-related prognostic signature high risk and IL-6 high expression were both associated with poor clinical outcome in NSCLC cisplatin-treated patients and increased infiltration of macrophages in TME. We provided evidence that cisplatin specifically upregulated IL-6 expression in MSCs via the MEK-ERK pathway to trigger macrophages recruitment to the TME. Thus, our study indicated that MSCs and IL-6 are rational targets for therapeutic intervention for NSCLC.

## Data Availability

The original contributions presented in the study are included in the article/Supplementary Material, further inquiries can be directed to the corresponding authors.
