# Supplementary material for: Mesenchymal stem cells promote cisplatin resistance in non-small cell lung cancer through IL-6/MEK-ERK/macrophages axis: construction of prognostic signature and experimental investigation
Source: Front Pharmacol. 2026 Apr 1;17:1793151. doi: 10.3389/fphar.2026.1793151 (PMC13080276; doi:10.3389/fphar.2026.1793151)
Supplement: Supplementary file 2 [file Supplementaryfile2.docx]

Supplementary Material


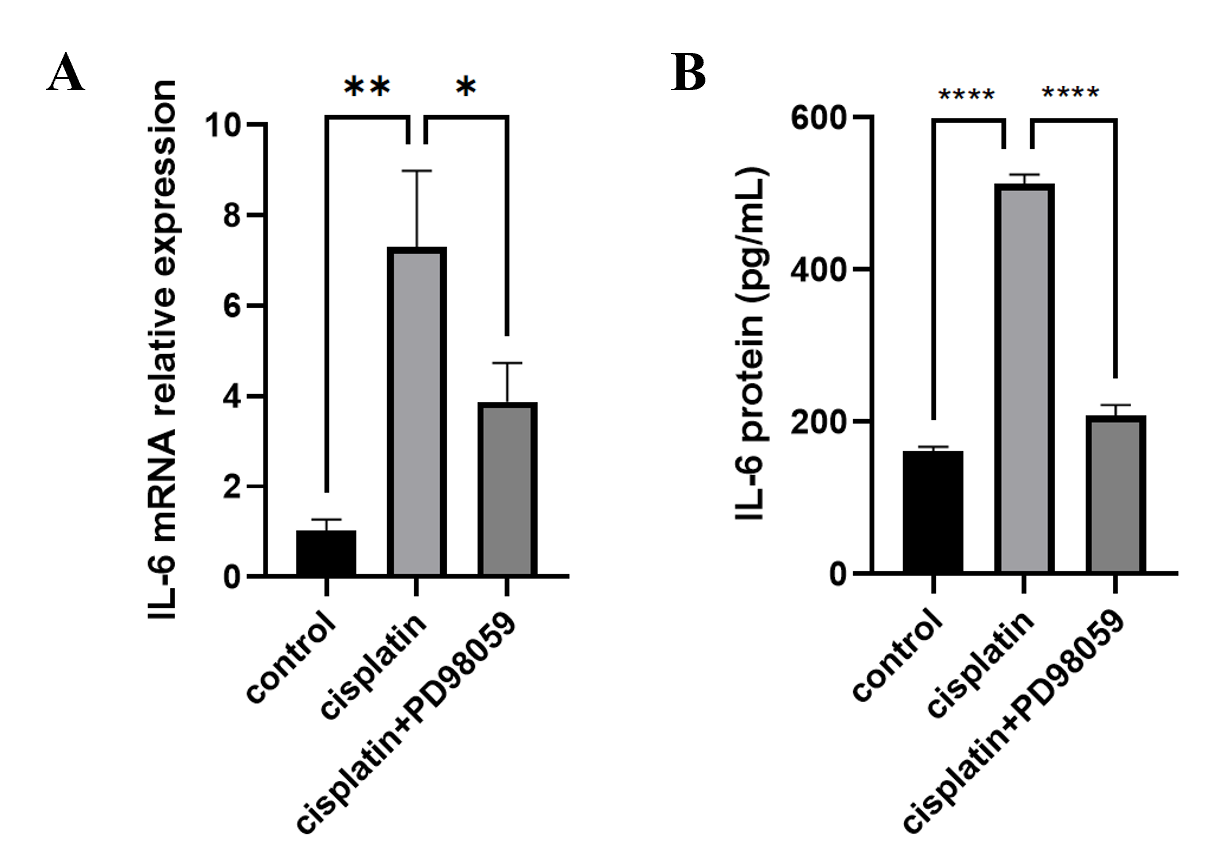


**Supplementary Figure 1.** Expression of IL-6 in human MSCs. **(A)** Expression of IL-6 mRNA at 24 h after treatment of cisplatin and PD98059. **(B)** ELISA results revealed IL-6 protein expression of MSCs at 24 h after treatment of cisplatin plus PD98059. *p < 0.05, **p < 0.01, ****p < 0.0001.
